# Single Agent Lenalidomide Activity in Multiple Myeloma Relapse Evidenced Uniquely by CT/PET

**DOI:** 10.4084/MJHID.2012.041

**Published:** 2012-06-18

**Authors:** Alessandro Gozzetti, Vania Rossi, Alfonso Cerase, Giulia Papini, Marzia Defina, Monica Bocchia

**Affiliations:** 1 Division of Hematology, Azienda Ospedaliera Universitaria Senese, Siena, Italy; 2 Unit of Nuclear Medicine, Ospedale San Donato, Arezzo, Italy; 3 Unit NINT Neuroimaging and Neurointervention, Department of Neurological and Sensorial Sciences, Azienda Ospedaliera Universitaria Senese, Policlinico “Santa Maria alle Scotte”, Siena, Italy

## Abstract

A 71 year old female with multiple myeloma presented with back pain seven year after autologous stem cell transplant. Skeletal bone survey and magnetic resonance imaging did not show a relapse that was evidenced by CT/PET. Lenalidomide as single agent induced a complete disappearance of the lesions 6 months later and confirmed after one year at CT/PET.

## Introduction

Multiple myeloma patients often develop osteolytic lesions that result in debilitating skeletal complications such as fractures, bone pain and hypercalcemia. The development of bone lesions is due to an uncoupled bone remodeling, i.e. the increased osteoclast bone resorption accompanied by a reduction in bone formation.^1^ The usual gold standard technique used at diagnosis is skeletal X-rays survey which is able to give information about radiolucent lesions but do not provide information about bone remodeling or small lesions. Magnetic resonance imaging (MRI) is able to detect smallest lesions present in the soft tissue around the bone and is widely used in the diagnostic process for the evaluation of the spinal cord and pelvis. One of the latest technique used in myeloma is positron emission tomography integrated with computed tomography (CT/PET) which detects with high sensitivity and specificity myeloma bone lesions.^2^ Moreover CT/PET has the advantage to demonstrate the presence of active disease and thus monitor response to therapy or even relapse.

Novel agents such as bortezomib, thalidomide and lenalidomide have given high rates of responses in multiple myeloma but their mechanisms of action are not fully understood and in particular their activity in bone disease is not fully elucitated. Lenalidomide has shown in vitro osteoclastic inhibition but to the best of our knowledge this effect has not been shown in vivo. We hereby describe a patient that after myeloma bone relapse evidenced uniquely by CT/PET had a complete disappearance of the lesions with lenalidomide therapy.

## Case Report

**Figure 1:**
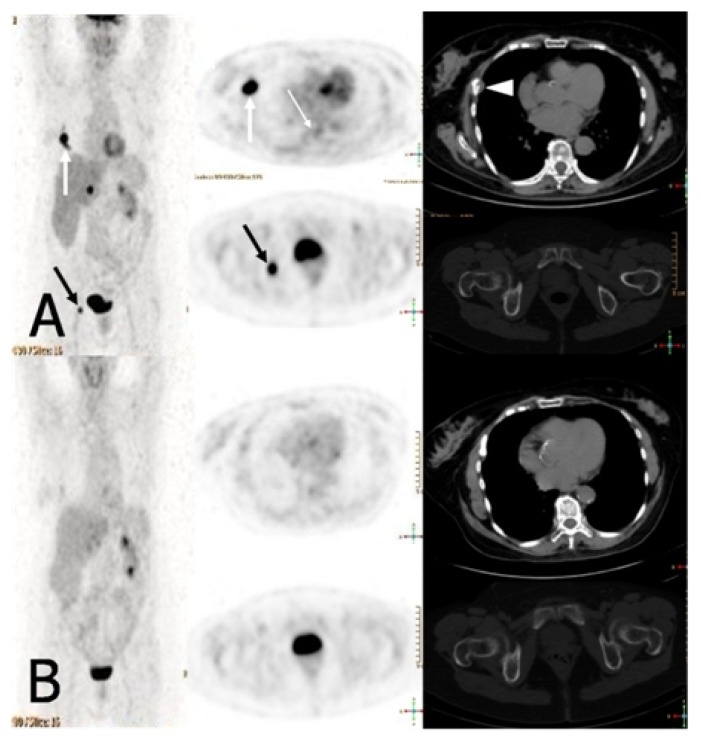
**A**. PET showing three focal lesions in the fifth right rib (*thick white arrows*), D10 vertebral body (*thin white arrow)*, and right ischium (*black arrow)*. Only the lesion of the fifth right rib was evident at CT images (*arrowhead)*. **B.** PET showing complete disappearance of the lesions after lenalidomide therapy.

A 71-year-old female with multiple myeloma (MM) IgG/lambda stage III A (II ISS) presented to routine follow-up seven years after 3 cycles of VAD (vincristine, adriamycin, dexamethasone) induction, ciclophosphamide and stem cell harvest followed by autologous stem cell transplantation (ASCT) conditioned with melphalan 200 mg/m^2^, complaining of a moderate pain in her right ribs and pelvis. Radiographs showed multiple stable osteolytic lesions in her spine, pelvis, ribs, and skull, when compared to those obtained 18-month before. An MRI of her spine confirmed the stability of bone lesions compared to the previous one done one year before. Bone marrow biopsy showed 10% of monoclonal plasma cells. Serum monoclonal component was stable (IgG 0.8 g/L). CT/PET using 18F labelled with Fluorodeoxyglucose (18F-FDG) as positron-emitting radionuclide (Figure 1 A) showed three hypermetabolic focal lesions in the fifth right rib (*thick white arrows*), D10 vertebral body (*thin white arrow)*, and right ischium (*black arrow)*, with standardized uptake value (SUV) of 14.8 (normal cut off 2.5), 3.3, and 9.3, respectively. Considering the moderate pain and in order to test unique lenalidomide efficacy in bone lesions, after signed written informed consent, the patient was treated uniquely by lenalidomide (25 mg/day, 1 to 21 every 28 days, for a total of 6 cycles). We avoided dexamethasone use on purpose, as well as bisphosphonates to show drug efficacy. Six-month follow-up PET-CT showed a complete disappearance of the lesions (Figure 1 B), that was confirmed one year later.

## Discussion

Imaging techniques have long been used to help diagnose patients and determine the stage of the disease. Nowadays they can be used to investigate also the response to treatment. Although MRI seems better than CT/PET in picking-up myeloma bone lesions, CT/PET is more effective in determine patient’s response. In our case, MRI was identical to a previous one done the year before, but CT/PET showed active focal disease in three distinct areas.

PET/CT involvement in myeloma patients has been demonstrated as a prognostic factor in a recent study in which involvement at diagnosis of at least 3 focal lesions, SUV > 4.2 and persistence of PET/CT positivity after autotransplant were poor prognosticators.

Immunomodulatory agents (IMiD’s) have become an important drug category in the treatment of MM. The agents have a complex mechanism of action that influences the microenvironment in the bone marrow. Clinical studies have confirmed that thalidomide reduces markers of bone resorption, while lenalidomide induces osteoclast arrest in myeloma patients through inhibition of PU.1 and pERK.^3^ However, they seem to have no effect on osteoblast exhaustion present in myeloma.

The interest of our case is twofold: to show usefulness of PET-CT in the clinical management of MM patients and to show by imaging the great efficacy of lenalidomide in MM bone lesions.
